# Combined Analysis of MicroRNAs and Target Genes Revealed miR156-SPLs and miR172-AP2 Are Involved in a Delayed Flowering Phenomenon After Chromosome Doubling in Black Goji (*Lycium ruthencium*)

**DOI:** 10.3389/fgene.2021.706930

**Published:** 2021-07-15

**Authors:** Shupei Rao, Yue Li, Jinhuan Chen

**Affiliations:** ^1^College of Biological Sciences and Technology, Beijing Forestry University, Beijing, China; ^2^National Engineering Laboratory for Tree Breeding, Beijing Forestry University, Beijing, China

**Keywords:** polyploid, florescence, miRNA, *Lycium ruthenicum*, molecular mechanism

## Abstract

Polyploidy, which is widely distributed in angiosperms, presents extremely valuable commercial applications in plant growth and reproduction. The flower development process of higher plants is essential for genetic improvement. Nevertheless, the reproduction difference between polyploidy and the polyploid florescence regulatory network from the perspective of microRNA (miRNA) remains to be elucidated. In this study, the autotetraploid of *Lycium ruthenicum* showed late-flowering traits compared with the progenitor. Combining the association of miRNA and next-generation transcriptome technology, the late-flowering characteristics triggered by chromosome duplication may be caused by the age pathway involved in miR156-SPLs and miR172-AP2, which inhibits the messenger RNA (mRNA) transcripts of *FT* in the leaves. Subsequently, FT was transferred to the shoot apical meristem (SAM) to inhibit the expression of the flowering integration factor *SOC1*, which can eventually result in delayed flowering time. Our exploration of the flowering regulation network and the control of the flowering time are vital to the *goji* producing in the late frost area, which provides a new perspective for exploring the intrinsic molecular mechanism of polyploid and the reproductive development of flowering plants.

## Introduction

The integrated lifecycle of higher plants experiences a series of developmental processes subsuming seed germination, vegetative growth, flowering, fertilization, embryonic development, and seed formation. Among them, flowering is a momentous physiological behavior and the symbol from vegetative to reproductive growth in the growth course of higher plants (Spanudakis and Jackson, 2014). Blooming in the punctual time point sets the stage for the successful sexual reproduction and subsequent seed and fruit development (Jung and Muller, 2009). Nevertheless, the regulation of flowering time, whose onset and progression are strictly controlled, is composed of an intricate gene network integrated with environmental and endogenous cues to control the expression of a group of crucial flowering genes in the shoot apical meristem (SAM) (Michaels, 2009; Lee et al., 2020). Florigen as a flowering hormone in plants is transmitted from the light-receiving organs to the SAM to form a floral response. Studies have proved that *CONSTANS* (*CO*) can induce *Flowering Locus T* (*FT*) expression in leaves. When both *CO* and *FT* were expressed in the SAM, only the *FT* can induce flowering in plants, which showed that the FT protein can transfer to the SAM to promote the flowering transformation and is the key component of florigen. *FT* is an important integration factor in various pathways of flowering, which promotes onset of the reproductive phase and flower formation and is further transmitted to downstream flowering development genes, thereby promoting flowering (Zhu et al., 2020). Insomuch as flowering time will noticeably affect plant health and crop yields, shifting the seasonal timing of reproduction is also the main goal of plant breeding. A meticulous grasp of the flowering time regulatory mechanisms, to produce new varieties that are better suitable for the change in the local climatic conditions, is essential for continuous improvement of agricultural practices.

Polyploidization has always been recognized as a crucial force and channel to facilitate plant evolution and speciation during the evolution process. The biological and genetic advantages of polyploid over diploid are tremendous. Most evidence have concluded that polyploid usually has novel characteristics that are unattainable by the diploid progenitor, such as increased organ biomass, resistance to adversity, and changes in flowering time, which may cause polyploidy to enter a new environmental niche and domesticate plants from a new perspective (Braynen et al., 2021). To better utilize the commercial worthlessness brought by polyploid, people often make use of the newly synthesized polyploid to simulate the polyploid generation process. A comparison of multiple biological genetics between the polyploid and the parent species in the existing research found that polyploid has undergone major changes in many aspects. At the moment, various scientific progresses in flowering transition has authenticated that many allotetraploid plants have a delayed flowering phenotype (Mayfield et al., 2011). Nevertheless, the pivotal factors that trigger polyploidy-induced late-flowering traits are ambiguous and complex. The influence not only exists in heritable variations, containing chromosome rearrangement and sequence elimination, but also in epigenetic variations, such as methylation, gene silencing, and small RNA changes.

Noncoding RNAs (ncRNAs) play an important role in regulating plant growth and development networks. With the emergence of deep sequencing technology, the research on sRNAs that cannot encode functional proteins has been accelerated. The structural categories of ncRNAs include ribosomal RNA (rRNA), transfer RNA (tRNA), small nuclear RNA (snRNA), and small nucleolar RNA (snRNA) (Brant and Hikmet, 2018). Regulatory ncRNAs are generally divided into long ncRNAs (lncRNAs > 200 nt) and small ncRNAs (sRNAs, 18–30 nt). sRNAs are mainly divided into two categories, namely, siRNA and microRNA (miRNA), which play an important role in gene regulation and plant development. siRNA is a nonendogenous double-stranded RNA, which can act on any part of mRNA and degrade target gene. Unlike siRNA, miRNA is a type of noncoding RNAs abundantly encoded by endogenous genes with a length of approximately 21–24 bases, which can precisely and effectively monitor gene expression at the posttranscriptional level through negative regulation methods comprising translation inhibition or degradation of the target gene mRNA (Song et al., 2019; Zhang et al., 2019a). Recently, the majority of previous research on miRNAs focuses on transcriptome analysis and function prediction, yet reports on the interaction of miRNAs in the reproductive biology of polyploid plants are relatively rare.

*Lycium ruthencium* is a traditional Chinese medicine of the Solanaceae and famous for its rich nutrients and medicinal value, which is mainly distributed in the late frost-prone areas in the northwestern region of China (Wang et al., 2018). *L. ruthenicum* contains water-soluble antioxidant proanthocyanidins and polysaccharides, which can reduce the production of reactive oxygen species (ROS) and inflammation and inhibit the growth of cancer cells (Zhang et al., 2019b). Geographically, the special climate of *L. ruthencium* growth zone is characterized by different degrees of frost every year, which is usually the most serious in spring. The transition from bud stage to flowering and fruiting stage of *L. ruthencium* occurs in spring. When the minimum temperature is lower than 0°C, the flower buds are likely to be frozen, dead, and withered, which directly leads to the decline of the yield and quality of the first fruit of *L. ruthencium*. However, the nutrient components of the first fruit are higher than those of the later summer and autumn fruits and occupy an important position in the wolfberry industry. Therefore, staggering the flowering and frost periods has a greater impact on the production and the development of the wolfberry industry. Here, we focused on the difference in the flowering time of *L. ruthencium* tetraploid and analyzed the molecular mechanism of tetraploid on the change in flowering period from the perspective of miRNA and transcriptome association analysis, in order to provide a theoretical basis for the creation of late-flowering.

## Materials and Methods

### Plant Growth Condition and Phenotype Identification in the Floral Organs of *Lycium ruthencium*

Diploid seeds of *L. ruthencium* from Xinjiang Province were disinfected with sodium hypochlorite and planted in sterile Murashige and Skoog (MS) medium for 30 days. The 4-week-old leaves were used for colchicine chromosome doubling into autotetraploid. The diploid and autotetraploid seedlings that had grown homogeneously were planted in a mixture of sterilized turfy soil and vermiculite at a ratio of 3:1 under a 16-h light/8-h dark cycle at a temperature of 24 ± 1°C in the greenhouse for 14 days. The flowering time of tetraploid and diploid flowers planted in Ningxia under consistent growth conditions was observed. About 0.2 g true leaves of different ploidy *L. ruthencium* was collected as sample for sequencing. We selected a typical individual to serve as a biological replicate. A total of three replicates were obtained. Six samples were immediately frozen in liquid nitrogen after harvest and stored at −80°C for further use.

### RNA Isolation and miRNA Sequencing

Total RNA was extracted using RNAprep Pure Plant Kit (TIANGEN Biotech Co., Ltd., Beijing, China) following the manufacturer’s recommendations. The RNA was monitored by 1% agarose gel electrophoresis for RNA quality, and the concentration was detected using NanoDrop 2000. After the ends of the RNA are ligated to the adaptor, the library was subjected to reverse transcription by reverse transcriptase using RT Primer. After PCR amplification on the library, polyacrylamide gel electrophoresis (PAGE) was used to recover a small RNA library of about 150 bp to generate a stable library for sequencing. The stable small RNA library was mixed, denatured, and added to the Illumina HiSeq X Ten sequencing platform (Illumina Inc., San Diego, CA, United States) for high-throughput single-read sequencing (Cao et al., 2018).

### Data Filtering and Statistics of sRNA

The reads of low-quality or contaminants sequence were filtered, and the reads containing polyA/polyT and outside the miRNA length range (15–34 nt) from high-throughput raw data were removed using cutadapt (Martin, 2011) to prevent the data quality and subsequent analysis being affected. Repeated reads were combined, and the distribution of reads in each sample was calculated. Based on clean reads small RNA (sRNA) sequence types, number and length distribution. The small RNA sequences were searched and annotated by BLASTN^1^, and the Rfam database^2^, which integrates ncRNA family sequences, can remove possible rRNA, tRNA, and snRNA with Bowtie (Langmead, 2009) and classify the small RNA annotated or predicted as mature miRNAs to analyze subsequent experiments.

### MiRNA Analysis

The remaining sRNA tags without rRNA, tRNA, snRNA, and snoRNA are compared with the mature miRNA sequences of other species through BLAST and miRbase (^3^, version 22) databases considering maximum two mismatches or gaps to identify the known mature miRNA in the sequencing data (Cagirici et al., 2021). Sequences that have not been identified as known miRNAs are mapped to the reference genome of homologous species by Bowtie 2, and then, the MIREAP^4^ was used to predict novel miRNAs, which was recognized by the defining characteristics such as the secondary structures (Langmead and Salzberg, 2012). Simultaneously, the miRNA count, length, and nucleotide bias at each position were calculated for each candidate novel miRNA.

### Analysis of Differentially Expressed miRNA

The reads number of each miRNA in all samples was analyzed statistically, and the expression was normalized by the reads per million (RPM) value to the same magnitude:

$$RPM=\frac{eachmiRNAreads}{allmiRNAreads}$$

The DEGseq R package was used to identify differentially miRNAs expression between the two samples based on the significant principle that expression | fold change (FC)| > 2 and *p* < 0.05. Statistically tested values were adjusted by the false discovery fate (FDR). Differential miRNA expression was visually displayed *via* volcano plot and cluster heatmap by scatter plot and MEV software.

### Target Prediction and Enrichment Analysis

miRanda and RNAhybrid as the classical algorithms for identifying sRNAs’ target loci were used for all miRNAs detected in this study. miRanda considers sequence complementarity, free energy of miRNA targets double strand, and cross-species conservation of target sites. RNAhybrid can be used to analyze the sequence complementarity, target site abundance, and minimum free energy (MFE). MFE ≤ -22 kcal/mol and *p* ≤ 0.1 were used for RNAhybrid analysis. The sequence complementarity score = 145 and energy = -10 kcal/mol were parameters for miRanda analysis (Ye et al., 2018). The target genes of whole differentially expressed miRNA were putatively predicted using miRanda and RNAhybrid, respectively, and the intersection was taken as the ultimate target prediction results. Gene Ontology (GO) is an internationally standardized gene function classification system that provides a dynamically updated standard vocabularies to comprehensively describe the attributes of genes and products in organisms. The GO term was taken as the unit and the hypergeometric test applied to find out the GO term with a *p* < 0.05 compared with all expression backgrounds and defined it as a GO term that is significantly enriched in the target gene.

### Validation of miRNA and Target mRNA Expression Using qRT-PCR

Total RNA was extracted using a plant polysaccharide polyphenol RNA kit (TIANGEN Biotech Co.). The miRNA RT/qPCR Detection Kit (Aidlab Biotechnologies Co., Ltd., Beijing, China) was employed to carry out the poly(A) tailing and reverse transcription reaction on the 3′ end of the extracted total RNA. The quantitative real-time PCR (qRT-PCR) analysis of transcriptome was performed using the 2 × SYBR^®^ Green qPCR Mix Kit (Aidlab Biotechnologies Co., Ltd., Beijing, China). A three-step method was performed for fluorescence quantitative PCR detection using an ABI PRISM 7500 Real-Time PCR system (Applied Biosystems, Foster City, CA, United States). The relative expression of interesting miRNA and DEGs was normalized by actin. The real-time PCR data were analyzed by the 2^–ΔΔ*CT*^ method.

## Results

### Autotetraploid Exhibits Delayed Flowering in *Lycium ruthencium*

To observe whether the flowering time of *L. ruthenicum* is different from that of diploid after chromosome duplication, we found a ubiquitous phenomenon existing in flowering plants; the floral transition-induced tetraploid was delayed about 7–10 days compared with diploids, which is the same as *L. ruthenicum*.

### Summary of Small RNAs in Different Ploidy Black *goji*

**FIGURE 1:**
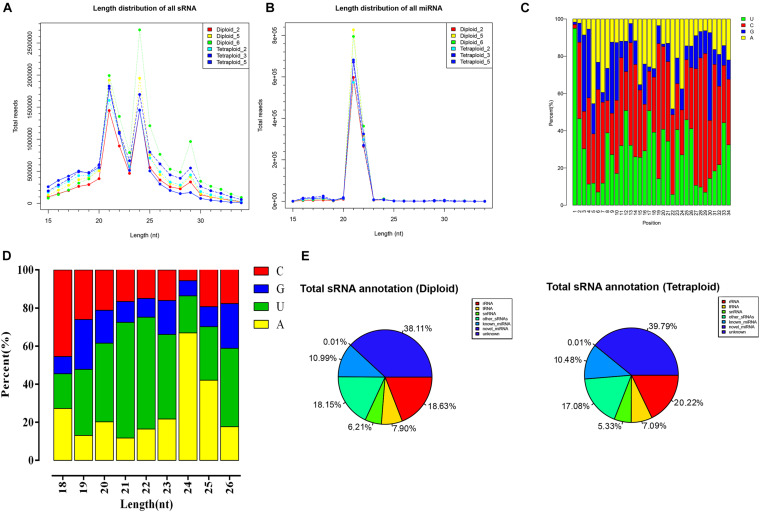
The length distribution and base preference of small RNAs in different ploidy *L. ruthenicum.* **(A,B)** The length distribution of sRNA and mRNA of different ploidy *L. ruthenicum.* **(C)** The base preference analysis of different positions of all miRNA. **(D)** The base preference analysis of first position in miRNA with 18–26 nt length. **(E)** The percentage graph of sRNA annotation status of each sample.

To explore the role of chromosomal replication events in the miRNA regulatory network of *L. ruthenicum*, six libraries of small RNA derived from diploid and autotetraploid leaves were sequenced (PRJNA727809). In summary, more than 9.0 × 10^6^ raw reads were drawn from six small RNA libraries performed by Illumina sequencing technology. After filtering low-quality sequences, more than 8.40 × 10^6^ high-quality clean reads were obtained, ranging from 8.40 × 10^6^ to 1.44 × 10^7^ for each sample, accounting for 91.80–94.90% (Table 1). Generally, the peaks of the length distribution can help us to determine the types of small RNAs, so as to make statistics on the types and quantity distributions of unique reads between two samples. Statistics on the length of small RNAs according to the sequencing results displayed that the lengths ranged from 15 to 34 nt, with peaks at 21 and 24 nt, and the most abundant miRNA length was mainly concentrated at 21 nt (Figures 1A,B). The base analysis of different positions of miRNA found that the different positions showed obvious bases preference. When miRNA precursors were transformed into mature ones, the specificity of the Dicer restriction site makes the first base of the mature sequence have a strong preference for U, and the occurrence probability is > 90% (Figure 1C). Analyzing the first position of miRNA with a length of 18–26 nt showed that, except for the total number of miRNA sequences of 18 nt, 19 nt, 25 nt, and 26 nt was too few to be statistically significant, the initiation preference for other miRNAs of different lengths had certain differences. The 20–23 nt miRNA is prone to U, especially that the probability of U base appearing in the first position of 21 nt length is as high as 60%, and the first base of miRNA of 24 nt is prone to appear to be A base (Figure 1D). From the perspective of sRNA classification and annotation, the number of unannotated small RNAs in all samples far exceeds the annotated small RNAs, and the rRNAs, with a determination rate remaining at about 19%, were the most abundant noncoding RNAs followed by other sRNAs, tRNA, and snRNA. The abundance of known miRNAs was higher than that of putative novel miRNAs (Supplementary Table 1 and Figure 1E).

**TABLE 1:** Statistics of small RNA sequencing in different ploidy samples of *L. ruthenicum.*

Sample	Raw Reads (M)	Raw Bases (G)	Clean Reads (M)	Clean Bases (G)	Clean Q20 (G)	Clean Q30 (G)
Diploid_2	9.158	1.383	8.406 (91.8%)	0.194 (14.0%)	0.194 (99.9%)	0.193 (99.5%)
Diploid_5	11.357	1.715	10.729 (94.5%)	0.246 (14.3%)	0.246 (99.9%)	0.245 (99.7%)
Diploid_6	15.199	2.295	14.423 (94.9%)	0.346 (15.1%)	0.346 (99.9%)	0.345 (99.7%)
Tetraploid_2	11.185	1.689	10.172 (90.9%)	0.233 (13.8%)	0.233 (99.9%)	0.232 (99.7%)
Tetraploid_3	12.821	1.936	11.876 (92.6%)	0.275 (14.2%)	0.275 (99.9%)	0.274 (99.6%)
Tetraploid_5	10.373	1.566	9.534 (91.9%)	0.209 (13.3%)	0.209 (99.9%)	0.208 (99.8%)

### Conserved and Novel miRNA Identification

**FIGURE 2:**
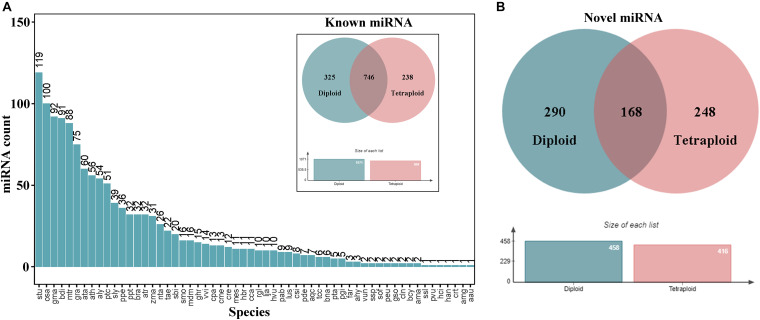
Species distribution and expression of different ploidy miRNAs. **(A)** The species distribution of all miRNAs. **(B)** The expression of known and novel miRNA in different ploidy *L. ruthenicum*.

To analyze miRNAs in subsequent experiments, we first compared the miRBase database and performed bioinformatics predictions to identify mature miRNAs and sorted out known and novel miRNAs. Considering that *L. ruthenicum* has no reference genome, whole plant miRNA sequences in miRbase have been extracted for comparison and quantitative analysis, and the results revealed that 646, 707, 807, 658, 655, and 659 known miRNAs were identified, respectively, totaling 1,309 miRNAs and 706 novel miRNAs (Table 2). There were 1,309 known miRNAs from 735 miRNA families, of which 604 families have only one member, accounting for 82.18%. The species distribution statistics of the identified known miRNAs illustrated that the types of the known miRNA distributed in *Solanum tuberosum* (Stu) are the most abundant, approximately 119 categories, indicating that the miRNA sequence of *L. ruthenicum* has the closest homology relationship with the *S. tuberosum* (Figure 2A). Novel miRNAs were expressed at a relatively low level compared with conserved miRNAs. A total of 248 and 290 novel miRNAs were specifically expressed only in *L. ruthenicum* tetraploid and diploid, respectively (Figure 2B).

**TABLE 2:** Classification of known and novel miRNAs in different ploidy black goji.

Type	Known miRNA	Novel miRNA	Total miRNA
Diploid_2	646	148	794
Diploid_5	707	184	891
Diploid_6	807	288	1095
Tetraploid_2	658	185	843
Tetraploid_3	655	187	842
Tetraploid_5	659	162	821
Total	1309	706	2015

### Differentially Expressed miRNAs

**FIGURE 3:**
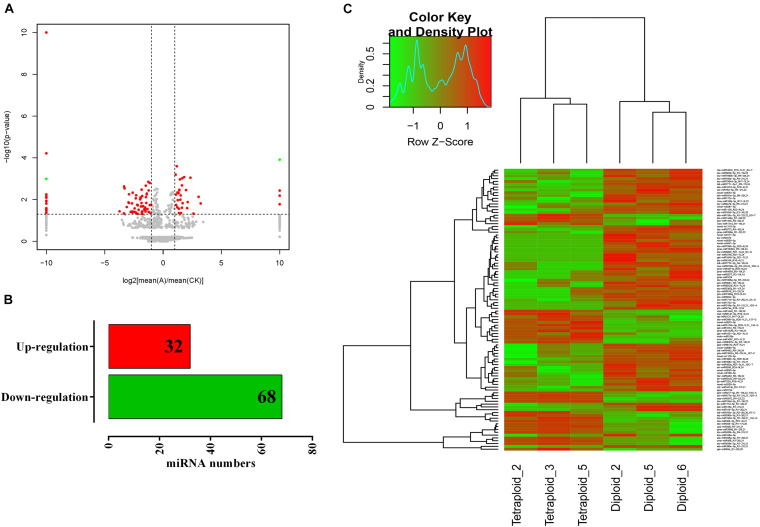
Differential miRNA screening and expression cluster analysis. **(A)** The volcano map of differentially expressed genes in diploid and tetraploid *L. ruthenicum*. **(B)** The number of up- and downregulated genes of differentially expressed genes. **(C)** The cluster heat map of differentially expressed genes. Green indicates the downregulation, and red indicates the upregulation.

For exploring the miRNA expression pattern after chromosome doubling in *Lycium*, the normalized miRNA expression levels of diploid and tetraploid plants were compared. A total of 100 differentially expressed miRNAs including 89 known and 11 novel miRNAs were screened to be significantly different among different ploidy plants according to the principle of | fold change (FC)| > 2 and *p* < 0.05 (Figure 3A). Thirty-two and 68 miRNAs play an up- and downregulated role in the growth and development of *L. ruthenicum*, indicating that the function of most miRNAs is inhibited after chromosome doubling (Figure 3B). Only novel m0925-5p was upregulated in the novel miRNAs, others were downregulated, and three of them even were not expressed in tetraploid (Supplementary Table 2). The gene expression trend reflects the potential function, and the miRNAs expression levels in all samples were statistically quantified and normalized using the fragments per kilobase per million (FPKM). Differentially expressed miRNAs were analyzed by hierarchical cluster analysis, in which miRNAs with the similar expression patterns were clustered. By clustering 100 differentially expressed miRNAs, the distribution statistics found that the differentially expressed miRNAs showed a variety of expression trends. For example, the different members of miR408 family were clustered together, showing the same trend; the expression level of three diploid varieties was extremely high, while that of tetraploid varieties was significantly lower (Figure 3C).

### Prediction and Annotation of miRNA Target Genes

Plants can exercise their regulatory function by the complementation of miRNA and mRNA. Based on the mechanism of plant miRNA, an efficient method to find the target gene of predicted miRNA and corresponding gene sequence is to use miRanda and RNAhybrid software. A total of 775 putative target genes were predicted *via* 100 differential miRNAs, of which 57 target genes corresponded to upregulated miRNAs, and 718 target genes were downregulated. Among the miRNAs mainly involved in the florescence regulation, miR156 and miR172 showed significant antagonistic expression, and the corresponding target genes were *SPLs* and *AP2*, respectively (Table 3).

**TABLE 3:** The differential miRNAs and target genes that affect the flowering time.

miRNA ID	miRNA family	Log_2_ (fold change)	Target gene
ata-miR156c-3p	miR156	1.485	Squamosa promoter-binding-like protein
bra-miR156d-3p		2.119	
stu-miR156d-3p		1.188	
mdm-miR156s		Inf	
ata-miR172c-3p	miR172	−1.528	APETALA 2
bra-miR172c-3p		−Inf	TOE3
ppe-miR172d		−Inf	

To determine the main biological functions performed by the target gene through the significant enrichment analysis of Gene Ontology (GO) and Kyoto Encyclopedia of Genes and Genomes (KEGG) function, GO/KEGG enrichment analysis was conducted on the predicted target genes, respectively. In GO enrichment analysis, there were 63 upregulated terms and 165 downregulated terms. A total of the most significant first 20 terms were intercepted, manifesting that the biological functions after chromosome doubling were gathered in the plant reproduction, including the development of floral organs and plant pollination (Table 4).

**TABLE 4:** The terms of GO enrichment by differential miRNA.

Category	Term	*p* value
GO:0006351	Transcription, DNA-templated	0.00004
GO:0003774	Motor activity	0.00006
GO:0006355	Regulation of transcription, DNA-templated	0.00008
GO:0051645	Golgi localization	0.00082
GO:0060151	Peroxisome localization	0.00082
GO:0014823	Response to activity	0.00093
GO:0032982	Myosin filament	0.00093
GO:0051646	Mitochondrion localization	0.00097
GO:0010321	Regulation of vegetative phase change	0.00117
GO:0006030	Chitin metabolic process	0.00118
GO:0080155	Regulation of double fertilization	0.00142
GO:0048653	Anther development	0.00152
GO:0009856	Pollination	0.0019
GO:0016459	Myosin complex	0.00199
GO:0042425	Choline biosynthetic process	0.00199
GO:0003677	DNA binding	0.00214
GO:0030048	Actin filament-based movement	0.00239
GO:0010468	Regulation of gene expression	0.00245
GO:0030016	Myofibril	0.00275
GO:0003700	Transcription factor activity…	0.00341

### qRT-PCR Validation of miRNA and mRNA Expression

**FIGURE 4:**
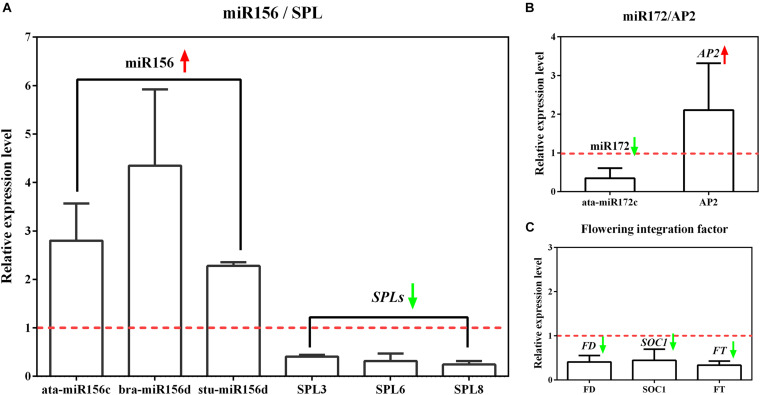
The relative expression of miRNA related to flowering time control and its target genes by qRT-PCR analysis. **(A)** The expression analysis of differential miR156 members and target gene SPL. **(B)** The expression analysis of differential miR172 members and target gene AP2. **(C)** The expression analysis of flowering integration factor FT, FD, and SOC1. The relative expression level represents the tetraploid compared to diploid under the premise that the expression level of diploid is equal to 1.

To prove the reliability and accuracy of miRNA sequencing, we selected the same samples as the sequencing to perform qRT-PCR verification on flowering-related miRNAs and several typical members of the corresponding target genes. The results of miRNA expression changes determined by qRT-PCR showed similar trends, indicating that the miRNA sequencing results are extremely accurate. In addition, the qRT-PCR identification of the target genes involved in the regulation of flowering time confirmed that the expression of these genes in tetraploid *L. ruthenicum* changed significantly (Figure 4).

## Discussion

Polyploid organisms generally display vigorous vitality and survivability, which is the reason why they easily produce in harsh environments such as glaciers and plateaus. Polyploid provides genomic deformability for the functional differences in duplicate gene, chromosome recombination, transcriptome changes, and gene dosage effects, which makes an important role in promoting species evolution (Van De Peer et al., 2017). As the outstanding abilities of polyploid are continuously excavated, breeders aim to attract the high-quality traits and artificially formulate polyploid germplasm, expecting to broaden the selectable population and promote the breeding industry development in China. Despite that the research location of polyploidy has been re-established, the molecular mechanism of isogeny polyploid traits with the same subgenome is still lacking.

Autotetraploids have been permanently created for many plant species in nature, including Salicaceae (Guo et al., 2017; Braynen et al., 2021), Cruciferae (Storme and Geelen, 2019), Zizyphus (Gu et al., 2005), and Actinidia (Wu et al., 2012). The majority of innovative traits formed by autotetraploid is mostly reflected in the gigantism caused by the genetic multiplication, the nutrients utilization, and the adversity adaptability. The research on the flowering time of autotetraploid is relatively scarce. In angiosperms, the regulation of floral organs and flowering period directly affects the fruits yield and the reproduction of offspring, which are vital to the life course of plants (Ionescu et al., 2017). In our study, we used the *L. ruthenicum* autotetraploid created in our laboratory, which exhibited a consistent phenotype the same as most tetraploids in vegetative growth, such as slower growth, larger and thicker leaves, and thicker stem (Rao et al., 2019). Compared with the first flowering period in spring with the diploid, it was found that flowering of tetraploid was universally delayed, indicating that chromosome doubling affects the vegetative growth and reproductive behavior of plants. The conclusion that chromosome doubling induced delayed flowering fully provides a reference for avoiding the decline of the first stubble *L. ruthenicum* fruit quality under the effect of late spring frost.

The continuous upgrading of high-throughput sequencing technology can achieve pivotal transcripts, which has become a broad-spectrum method for exploring plant biomolecules (Ma et al., 2015). The expression of miRNAs has been found to be altered in different plant growth and adverse circumstances, which helps shed light on plant internal mechanism from a new perspective (Ferdous et al., 2015; Muslu et al., 2021). We applied high-throughput sequencing to consider how chromosome doubling influences the florescence of *L. ruthenicum* at the miRNA level and eventually got the transcript and miRNA information of the autotetraploid and diploid ancestor. A total of six sRNA complementary DNA (cDNA) libraries of different ploidy *L. ruthenicum* were obtained, and more than 8.40 × 10^6^ high-quality clean reads were constructed. To study miRNA function, it is important to directly find the corresponding regulated target genes. Based on libraries screening, 100 differentially expressed miRNAs and 775 putative target genes of different ploidy were obtained according to the FDR standard, confirming that when the chromosome ploidy changes, the miRNA expression level in plants will have a tremendous change. The selected miRNAs were mainly concentrated in the plant reproduction by GO enrichment, including the development of floral organs and plant pollination, which further demonstrates that miRNAs play a vital role in chromosome doubling to regulate the delayed flowering phenotype of *L. ruthenicum*.

Some of the multitudinous screened differential miRNAs are significantly expressed and have momentous significance in the flowering regulation, such as miR156 and miR172. miR156 and miR172 belong to the miRNA family of extremely conserved and functional homeostasis. The expression of miR156 and miR172 in tetraploid compared with diploid showed a trend that the former was significantly upregulated and the latter was significantly downregulated. miR156 and miR172 manifested antagonistic regulation in *Arabidopsis* and some xylophyta plants. The former delayed flowering, and the latter promoted flowering, indicating that miR156 and miR172 are the fundamental miRNA families affecting late flowering of tetraploid. Researchers found that miR156 and target genes are key regulators to mediate the age pathway, and the content resolves the plants physiological age (Roussin-Leveillee et al., 2020). miR156 mainly regulates the physiological processes of plant growth, morphogenesis, flowering phase, secondary metabolism, and stress response through the SQUAMOSA PROMOTER BINDING PROTEIN-LIKE (SPL) transcription factor. SPL is a kind of plant-specific transcription factor that plays an important regulatory role in plant growth and development and also is a regulatory hub in the process of flower development (Wang et al., 2009). The expression of most SPLs is regulated by miR156/157 and has corresponding recognition sites. miRNA and transcriptome association analysis showed that miR156 and miR172 mainly targeted SPL3 and AP2 transcription factors. SPL3 can promote the mRNA expression of FT by combining with the GTAC element on the FT promoter and complete the transition from vegetative growth to reproductive growth (Jung et al., 2016). miR172 controls flowering time and organ formation by targeting AP2 transcription factor as FT repressor (Li et al., 2019). The protein product encoded by the FT gene is a flower-forming hormone that can be transported over long distances and is an important integration factor for the regulation of plant flowering. As a mobile flower formation signal, FT can combine with FLOWERING LOCUS D (FD) in the shoot apex meristem to form an FT–FD complex. The FT–FD dimer upregulates the expression of SOC1 gene and completes flowering induction (Smith et al., 2011).

**FIGURE 5:**
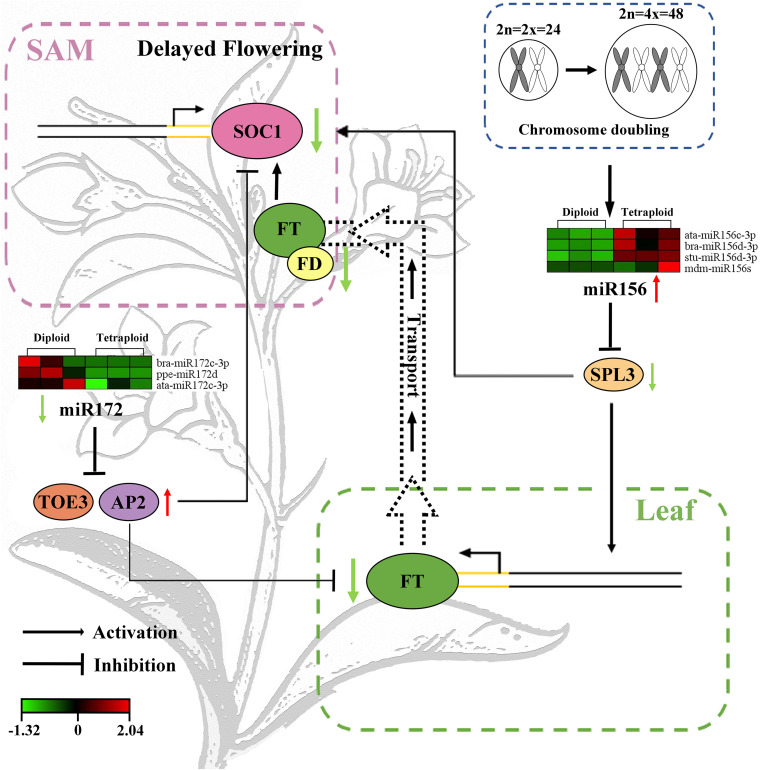
Molecular mechanism model of flowering delay regulated by chromosome doubling in *L. ruthenicum*. The green and red colors indicate low and high expression level, respectively. The left and right columns indicate diploid and tetraploid, respectively.

To summarize, the mechanism model of chromosome doubling induced late flowering of *L. ruthenicum* by participating in the age pathway involved in miR156-SPLs and miR172-AP2, which inhibited the mRNA expression of FT in the leaves. Subsequently, FT was transferred to the SAM to inhibit the expression of the flowering integration factor SOC1, which can eventually bring about the delay of flowering time (Figure 5). The exploration of mechanism provided the new available means for the regulation of the flowering period in polyploidy species, deepened the basic research of the *goji* industry, and may directly affect the crops yield.

## Data Availability Statement

The datasets presented in this study can be found in online repositories. The names of the repository/repositories and accession number(s) can be found below: NCBI (accession: PRJNA727809).

## Author Contributions

JC designed the experiments. SR performed the experiments and analyzed the data. JC and SR wrote the manuscript. JC and YL revised the manuscript. All authors read and approved the manuscript.

## Conflict of Interest

The authors declare that the research was conducted in the absence of any commercial or financial relationships that could be construed as a potential conflict of interest.

## Footnotes

1 http://blast.ncbi.nlm.nih.gov/Blast.cgi

2 http://www.sanger.ac.uk/software/Rfam

3 http://www.mirbase.org/

4 http://soxircefoige.net/projects/mireap/

## Supplementary Material

Supplementary Table 1 The relative abundance of each small RNA annotation in black goji.

Supplementary Table 2 The fold change of all miRNAs between diploid and tetraploid.
